# Dietary fat types consumption association with obesity and coronary indices

**DOI:** 10.1017/jns.2023.92

**Published:** 2023-11-03

**Authors:** Islam Al-Shami, Anfal Al-Dalaeen, Buthaina Alkhatib, Lana M. Agraib

**Affiliations:** 1 Department of Clinical Nutrition and Dietetics, Faculty of Applied Medical Sciences, The Hashemite University, Zarqa, Jordan; 2 Department of Clinical Nutrition and Dietetics, Faculty of Allied Medical Sciences, Applied Science Private University, Amman, Jordan; 3 Department of Food Science and Nutrition, Faculty of Agriculture, Jerash University, Jerash, Jordan

**Keywords:** Cholesterol, Coronary indices, MUFA, Obesity indices, PUFA, SFA, AIP, atherogenic index of plasma, AMDR, acceptable macronutrients distribution range, ATP, adenosine triphosphate, AVI, abdominal volume index, BAI, body adiposity index, BMI, body mass index, BRI, body roundness index, Chol, cholesterol, CI, conicity index, CMI, cardiometabolic index, CVD, cardiovascular disease, HDL, high-density lipoprotein cholesterol, LAP, lipid accumulation product, MetS, metabolic syndrome, MUFA, monounsaturated fat, NCD, non-communicable disease, PUFA, polyunsaturated fat, SFA, saturated fat, SPSS, Statistical Package for the Social Sciences, T2DM, type 2 diabetes, WC, waist circumference, WHtR, waist-to-height ratio, WWI, weight-adjusted-waist index

## Abstract

This article aims to study the different dietary fat types associated with obesity and coronary indices. A sample of 491 healthy adults was included in a cross-sectional manner. Dietary fats intake, obesity indices (conicity index (CI), body adiposity index (BAI), abdominal volume index (AVI), body roundness index (BRI), and weight-adjusted-waist index (WWI)), and cardiovascular indices (cardiometabolic index (CMI), lipid accumulation product (LAP), and atherogenic index of plasma (AIP)) were calculated and studied. Participants with an acceptable intake of omega-3 had a higher BRI score (1⋅90 ± 0⋅06 *v.* 1⋅70 ± 0⋅06). Participants with an unacceptable intake of cholesterol had a higher CI (1⋅31 ± 0⋅11 *v.* 1⋅28 ± 0⋅12; *P* = 0⋅011), AVI (20⋅24 ± 5⋅8 *v.* 18⋅33 ± 6⋅0; *P <* 0⋅001), BRI (2⋅00 ± 1⋅01 *v.* 1⋅70 ± 1⋅00; *P* = 0⋅003), WWI (11⋅00 ± 0⋅91 *v.* 10⋅80 ± 0⋅97; *P* = 0⋅032), and lower AIP (0⋅46 ± 0⋅33 *v*. 0⋅53 ± 0⋅33; *P* = 0⋅024). Total fat, saturated fat (SFA), and polyunsaturated fat (PUFA) intake had a significant moderate correlation with AVI and BRI. The monounsaturated fat (MUFA) intake had a significantly weak correlation with CI, AVI, BRI, WWI, and AIP. Cholesterol and omega-6 had weak correlations with all indices. Similar correlations were seen among male and female participants. The different types of fat intake significantly affected obesity and coronary indices, especially SFA and PUFA, as well as omega-3 and cholesterol. Gender and the dietary type of fat intake have a relationship to influence the indicators of both obesity and coronary indices.

## Highlights

1. The different types of fat intake significantly affected obesity and coronary indices.
2. SFA and PUFA, omega-3, and cholesterol had the most effect on obesity and coronary indices.
3. Gender and the dietary type of fat intake influenced obesity and cardiovascular indices.
4. Considering the fat type is more important than the amount.

## Introduction

Obesity has become a global public health concern due to its growing trends and unfavourable effects on health. It is a well-known risk factor for non-communicable diseases (NCDs), including hypertension, type 2 diabetes (T2DM), cardiovascular disease (CVD), and some malignancies.^(1)^ Traditionally, obesity is defined by body mass index (BMI). Age, sex, ethnicity, and muscle mass can influence the association between BMI and body fat. Moreover, BMI does not indicate how fat is distributed among people or distinguish between extra fat, muscle, or bone mass.^(2)^ Thus, researchers have explored several anthropometric indices to improve the above limitations. Many novel obesity indices were superior to the commonly used measurements by a wide range of recent epidemiological research that reported their high power in predicting the NCD's incidence or development risk.^(3–7)^ These indices included conicity index (CI), body adiposity index (BAI), abdominal volume index (AVI), body roundness index (BRI), and weight-adjusted-waist index (WWI).^(3–7)^

Specifically, BRI showed superior predictability and a strong correlation with the number of cardiometabolic risk variables.^(7)^ Higher BRI is associated with an increased risk of heart failure and T2DM.^(8,9)^ Also, a positive association between BRI and CI with selected cardiovascular risk factors, including insulin resistance, metabolic syndrome (MetS), hypertension, and dyslipidemia,^(4–6,10)^ while BRI and BAI were able to predict high metabolic risk.^(7,11)^ Likewise, AVI is the best for the discrimination of MetS.^(4,12)^ Besides, WWI is a special adiposity indicator exhibiting a positive linear relationship with cardiometabolic morbidity and mortality.^(3)^

Additionally, coronary indices have been used to detect and predict individuals with a high risk of developing cardiometabolic diseases worldwide. Of these, indices are cardiometabolic index (CMI), lipid accumulation product (LAP), and atherogenic index of plasma (AIP).^(13,14)^ Abolnezhadian and colleagues (2020) found that higher AIP, LAP, and CMI levels were related to higher insulin resistance, dyslipidemia, hypertension, and an inflammatory profile.^(13)^ Moreover, AIP was connected to blood sugar, obesity indicators, and blood lipid profiles, indicating that it may predict CVD.^(15)^

Excess fat intake is associated with increased obesity.^(1,16)^ So, for more than 40 years, to control obesity, dietary guidelines have focused on reducing total fat intake.^(1,17)^ Epidemiological research revealed that the consumption of particular types of fat has been linked to obesity and a wide range of diseases and adverse health outcomes, such as CVD, stroke, T2DM, and certain types of cancers.^(18–20)^ A negative association between polyunsaturated fatty acids (PUFA) and total cholesterol (Chol) consumption, BMI, and total fat mass has been reported.^(16)^ In addition, a low intake of omega-3 was found to be associated with increased obesity risk.^(21)^ On the other hand, reducing the intake of saturated fatty acids (SFA), increasing the consumption of monounsaturated fatty acids (MUFA), and a balanced intake of omega-3 and omega-6 to reduce the CVD risk factors are recommended.^(22)^

Hence, NCDs are a critical health obstacle in all Eastern Mediterranean Region countries. Jordan witnessed a significant increase in the prevalence of nearly all the risk factors involved in NCDs, causing morbidity and mortality.^(1)^ Among these risk factors, an unhealthy diet is the most significant, which is defined as a diet rich in SFA and Chol and is also low in fibre and nutritious food (fruit, vegetables, legumes, nuts, and grains).^(23)^ So, the current study aims to identify the impact of the type of consumed fat on obesity and coronary indices among Jordanian adults.

## Material and methodology

### Study design and approval

The data collection of this cross-sectional study was conducted on 491 healthy adults (who did not suffer from any diseases or have any signs and symptoms) from both genders aged 20 years and older and were randomly selected from Jordanian employees in the governmental universities. Using online Raosoft software (Raosoft, Inc. free online software, Seattle, WA, USA), the appropriate sample size was estimated to be 430 with a 5 % confidence interval, a 95 % confidence level, and a 50 % response distribution. The total number of participants reached 491, and they all were included. Female subjects who were pregnant or lactating, as well as those with mental illnesses, were all excluded from the study. People who had recent abdominal surgery that would have affected anthropometric or intra-abdominal measurements were also excluded. Before participation, each willing and qualified subject provided written, informed consent for the study inclusion. This study was conducted following the Declaration of Helsinki, as well as the protocol, and all of the procedures were approved by the Institutional Review Board (IRB) at The Hashemite University, Zarqa, Jordan (No.7/13/2020/2021).

### Data collection and analysis

Well-trained examiners gathered anthropometric measurements by following defined protocols. Participants’ body weight and height were recorded to the nearest 0⋅1 kg and 0⋅1 cm, respectively, while they were only wearing minimal clothing and bare feet. A flexible anthropometric tape was used to measure the participants’ waist circumference (WC) while they were standing. This measurement was made on the horizontal axis halfway between the lowest rib and the iliac crest. At the place where the buttocks are at their widest, the hip circumference (HC) was measured over thin clothing. The measurements of both circumferences were made to the nearest 0⋅1 cm. At the end of the interviews, appointments were scheduled to collect the participants’ blood samples for biochemical tests and they were instructed to fast for 12 h. To prepare the blood samples for analysis, they were centrifuged, separated, and kept at 4 °C. Five anthropometric (CI, BAI, AVI, BRI, and WWI) and three coronaries (CMI, LAP, and AIP) indices were calculated following their standard measuring formulas.^(3,11,13,24–26)^ Following are the lists of mathematical formulas that were used to calculate the obesity indices and coronary indices:

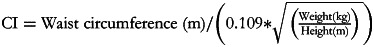
^(27)^
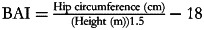
^(9)^
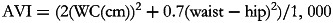
^(25)^
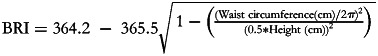
^(25)^
- WWI = (waist circumference (cm))/(weight (kg))^0.5^^(28,29)^
- AIP = Log(TG(mmol/L)/HDL-C(mmol/L))^(30)^
- LAP index for males = TG(mmol/L) × (waist(cm) − 65)^(31)^
- LAP index for females = TG(mmol/L) × (waist(cm) − 58)^(31)^
- CMI = TG/HDL-C × (waist-to-height ratio)^(32)^

Moreover, participants were asked to schedule another meeting, where participants came to the centre after fasting for 12 h to collect their blood samples to analyse the triglyceride (TG) and high-density lipoprotein (HDL). Also, two non-consecutive 24-h daily recalls were collected (weekday and weekend). Participants were asked to remember and mention all foods and beverages they consumed in the last 24 h, including preparation techniques and names of traditional and frequently used food brands, as well as memorise the nearest estimate for the consumed amount. The dietary intake of total fat and fat types (total fat, SFA, Chol, MUFA, PUFA, omega-3, and omega-6) was calculated using ESHA's Food Processor®, Nutrition Analysis Software (version 11:0; ESHA Research). Based on the recommendations of the US Institute of Medicine (IOM), adequate consumption of SFA, MUFA, PUFA, Chol, omega-3, and omega-6 was considered when the ingestion was <10, ≤20, and ≤10 % of the daily energy value and <300 mg/d, >1⋅6 g/d, and 17 g/d, respectively.^(33)^ However, the total fat consumption was found to be acceptable when compared to the ‘acceptable macronutrient distribution ranges (AMDRs)’ for healthy adults (AMDRs = 20–35 % of total daily energy intake).^(33)^

### Statistical analysis

Statistical Package for the Social Sciences (SPSS) version 25 (IBM, Chicago, IL, USA) was used to perform all the statistical analyses of data. The normality of the variables was examined using the Shapiro–Wilk test. Means and standard deviation to present the continuous variables were calculated using an independent *t*-test. Frequencies and percentages to present the categorical variables were calculated using Chi-square tests to describe the sample. Pearson correlation was performed to determine the correlation between obesity and coronary indices and different types of fat intake. The findings were considered statistically significant when the *P*-value was <0⋅05.

## Results

The table of general characteristics is published elsewhere.^(34)^ This study examined 491 healthy adults in total, 344 males and 147 females. The male participants had a mean body weight of 81⋅91 ± 14⋅76 kg, BMI of 27⋅49 ± 4⋅80 kg/m^2^, WC of 99⋅36 ± 13⋅67 cm, and HC of 105⋅17 ± 9⋅17 cm. The female participants had a mean body weight of 70⋅61 ± 16⋅19 kg, BMI of 27⋅94 ± 6⋅61 kg/m^2^, WC of 88⋅75 ± 15⋅19 cm, and HC of 106⋅05 ± 13⋅54. Male participants were mainly between 20 and 34 years (38⋅1 %) and 35 and 44 years (33⋅4 %). Similarly, female participants were between 20 and 34 (45⋅6 %) and 35 and 44 (36⋅7 %). Female participants had a higher prevalence of university education (76⋅9 %), while male participants had a higher prevalence of school education (58⋅4 %). Most male or female participants were married (76⋅7 and 60⋅5 %, respectively), with an income range of 200–499 Jordanian dinars (JD)s (54⋅4 and 57⋅8 %, respectively). 53⋅2 % of the male participants were smokers. At the same time, 51⋅0 and 36⋅7 % of female participants were non-smokers and passive smokers, respectively. Most male participants were physically active (61⋅9 %), while female participants were physically inactive (53⋅7 %). Virtually all of the study participants had no history of chronic diseases.

Table 1 shows the adherence for recommended percent intake from various types of fats and for total fat consumption among the study population stratified by gender. The total fat percentage's mean was about 39 % (39⋅5 % for males and 37⋅4 % for females) higher than the AMDR's upper limit. Furthermore, only 30⋅8 % of males and 33⋅3 % of females consumed the recommended total fat (AMDRs: 20–35 %). Moreover, male participants had a significantly higher prevalence of an acceptable MUFA intake than females (53⋅2 % *v*. 42⋅9 %, respectively). Among recommended intake from different types of fat, 44⋅2 % of male participants and 30⋅6 % of female participants had significantly higher Chol intake (*P* = 0⋅005). Although there were no significant differences between both genders, most male and female participants had an unacceptable intake of PUFA (79⋅4 and 68⋅0 %, respectively) and omega-3 fatty acids (96⋅2 and 97⋅3 %, respectively). Table 1 also demonstrates the mean and sd for the acceptable and unacceptable values for each type of fat as well as for the total fat percent regarding the IOM and AMDR's guidelines.

**Table 1.** Gender-specific fat consumption according to recommendations among the study population

Fat types (Mean ± sd)	Gender		*P*-value*
	Male (*n* 344)	Female (*n* 147)	
Fat			
Unacceptable (43⋅5 ± 7⋅6 %)	238 (69⋅2)	98 (66⋅7)	0⋅582
Acceptable (29⋅0 ± 3⋅9 %)	106 (30⋅8)	49 (33⋅3)	
Saturated fatty acids			
Unacceptable (12⋅1 ± 2⋅1 %)	164 (47⋅7)	78 (53⋅1)	0⋅274
Acceptable (7⋅1 ± 1⋅5 %)	180 (52⋅3)	69 (46⋅9)	
MUFA			
Unacceptable (10⋅6 ± 3⋅1 %)	161 (46⋅8)	84 (57⋅1)	0⋅036
Acceptable (21⋅7 ± 5⋅3 %)	183 (53⋅2)	63 (42⋅9)	
PUFA			
Unacceptable: 5⋅6 ± 2⋅3 %	273 (79⋅4)	100 (68⋅0)	0⋅007
Acceptable: 13⋅3 ± 2⋅9 %	71 (20⋅6)	47 (32⋅0)	
Cholesterol			
Unacceptable (470⋅5 ± 174⋅5 mg)	152 (44⋅2)	45 (30⋅6)	0⋅005
Acceptable (172⋅0 ± 76⋅3 mg)	192 (55⋅4)	102 (69⋅4)	
Omega-3 fatty acids			
Unacceptable (0⋅96 ± 0⋅41 g)	331 (96⋅2)	143 (97⋅3)	0⋅557
Acceptable (2⋅88 ± 1⋅31 g)	13 (3⋅8)	4 (2⋅7)	
Omega-6 fatty acids			
Unacceptable (9⋅49 ± 4⋅74 g)	158 (45⋅9)	64 (43⋅5)	0⋅626
Acceptable (32⋅97 ± 18⋅27 g)	186 (54⋅1)	83 (56⋅5)	

Acceptable value was considered when fat % of total energy intake = 20–35 %, SFA < 10 %, MUFA ≤ 20 %, PUFA ≤ 10 %, Chol < 300 mg/d, omega-3 > 1⋅6 g/d, and omega-6 = 17 g/d. However, other than these reference values were considered unacceptable.

MUFA, monounsaturated fatty acids; PUFA, polyunsaturated fatty acids.

* *P*-value < 0⋅05 considered statistically significant (two-tailed).

The mean scores of obesity and coronary indices were not significantly different through the recommended intake of the other fat types categories, except for BRI among omega-6 recommended intake where the participant in acceptable intake had a higher BRI score (1⋅90 ± 0⋅06) compared to unacceptable intake participants (1⋅70 ± 0⋅06) (Table 2). Regarding Chol, participants in the unacceptable intake group were found to have significantly higher mean scores of CI (1⋅31 ± 0; *P* = 0⋅011), AVI (20⋅24 ± 5⋅8; *P <* 0⋅001), BRI (2⋅00 ± 1⋅01; *P* = 0⋅003), WWI (11⋅00 ± 0⋅91; *P* = 0⋅032), and lower AIP (0⋅46 ± 0⋅33; *P* = 0⋅024) when compared to participants in the acceptable intake group (CI 1⋅28 ± 0⋅12; AVI 18⋅33 ± 6⋅0; BRI 1⋅70 ± 1⋅00; WWI 10⋅80 ± 0⋅97; and AIP 0⋅53 ± 0⋅33).

**Table 2.** The mean score of obesity and coronary indices through the recommended intake from different fat types categories (*n* 491)

Fat type	CI	BAI	AVI	BRI	WWI	CMI	LAP	AIP
Fat								
Unacceptable	1⋅29 ± 0⋅12	−17⋅50 ± 0⋅09	19⋅09 ± 6⋅03	1⋅81 ± 1⋅04	10⋅87 ± 0⋅99	2⋅42 ± 2⋅52	56⋅47 ± 49⋅7	0⋅48 ± 0⋅32
Acceptable	1⋅30 ± 0⋅11	−17⋅50 ± 0⋅09	19⋅11 ± 5⋅83	1⋅80 ± 0⋅94	10⋅90 ± 0⋅86	2⋅86 ± 4⋅11	67⋅6 ± 111⋅26	0⋅51 ± 0⋅36
*P*-value*	0⋅724	0⋅889	0⋅964	0⋅894	0⋅752	0⋅144	0⋅126	0⋅385
Saturated fatty acids								
Unacceptable	1⋅30 ± 0⋅01	−17⋅51 ± 0⋅01	18⋅90 ± 0⋅40	1⋅79 ± 0⋅07	10⋅90 ± 0⋅06	2⋅72 ± 0⋅25	62⋅57 ± 6⋅15	0⋅48 ± 0⋅02
Acceptable	1⋅29 ± 0⋅01	−17⋅50 ± 0⋅01	19⋅28 ± 0⋅36	1⋅83 ± 0⋅06	10⋅85 ± 0⋅06	2⋅41 ± 0⋅13	57⋅47 ± 2⋅96	0⋅50 ± 0⋅02
*P*-value*	0⋅755	0⋅165	0⋅48	0⋅61	0⋅587	0⋅277	0⋅451	0⋅661
MUFA								
Unacceptable	1⋅30 ± 0⋅01	−17⋅50 ± 0⋅01	19⋅14 ± 0⋅39	1⋅83 ± 0⋅07	10⋅89 ± 0⋅06	2⋅69 ± 0⋅24	61⋅85 ± 5⋅97	0⋅49 ± 0⋅02
Acceptable	1⋅30 ± 0⋅01	−17⋅51 ± 0⋅01	19⋅05 ± 0⋅37	1⋅79 ± 0⋅06	10⋅86 ± 0⋅06	2⋅43 ± 0⋅14	58⋅12 ± 3⋅20	0⋅49 ± 0⋅02
*P*-value*	0⋅993	0⋅405	0⋅877	0⋅654	0⋅772	0⋅344	0⋅582	0⋅941
PUFA								
Unacceptable	1⋅29 ± 0⋅01	−17⋅51 ± 0⋅00	18⋅91 ± 0⋅28	1⋅77 ± 0⋅05	10⋅86 ± 0⋅05	2⋅63 ± 0⋅16	61⋅11 ± 4⋅16	0⋅51 ± 0⋅02
Acceptable	1⋅30 ± 0⋅01	−17⋅49 ± 0⋅01	19⋅68 ± 0⋅68	1⋅95 ± 0⋅12	10⋅93 ± 0⋅11	2⋅33 ± 0⋅27	56⋅40 ± 4⋅95	0⋅44 ± 0⋅03
*P*-value*	0⋅792	0⋅097	0⋅221	0⋅091	0⋅468	0⋅356	0⋅552	0⋅08
Cholesterol								
Unacceptable	1⋅31 ± 0⋅11	−17⋅51 ± 0⋅09	20⋅24 ± 5⋅8	2⋅00 ± 1⋅01	11⋅00 ± 0⋅91	2⋅73 ± 2⋅31	56⋅56 ± 87⋅7	0⋅46 ± 0⋅33
Acceptable	1⋅28 ± 0⋅12	−17⋅49 ± 0⋅08	18⋅33 ± 6⋅0	1⋅70 ± 1⋅00	10⋅80 ± 0⋅97	2⋅45 ± 3⋅55	65⋅08 ± 49⋅9	0⋅53 ± 0⋅33
*P*-value*	0⋅011	0⋅219	<0⋅001	0⋅003	0⋅032	0⋅33	0⋅217	0⋅024
Omega-3 fatty acids								
Unacceptable	1⋅29 ± 0⋅01	−17⋅50 ± 0⋅00	19⋅06 ± 0⋅27	1⋅80 ± 0⋅05	10⋅87 ± 0⋅04	2⋅58 ± 0⋅14	60⋅06 ± 3⋅48	0⋅49 ± 0⋅02
Acceptable	1⋅31 ± 0⋅03	−17⋅50 ± 0⋅02	20⋅09 ± 1⋅66	1⋅94 ± 0⋅26	11⋅02 ± 0⋅26	2⋅06 ± 0⋅35	57⋅87 ± 11⋅10	0⋅43 ± 0⋅07
*P*-value*	0⋅504	0⋅93	0⋅485	0⋅591	0⋅524	0⋅498	0⋅906	0⋅447
Omega-6 fatty acids								
Unacceptable	1⋅29 ± 0⋅01	−17⋅51 ± 0⋅01	18⋅54 ± 0⋅38	1⋅70 ± 0⋅06	10⋅83 ± 0⋅06	2⋅39 ± 0⋅14	56⋅53 ± 3⋅33	0⋅49 ± 0⋅02
Acceptable	1⋅30 ± 0⋅01	−17⋅50 ± 0⋅01	19⋅55 ± 0⋅37	1⋅90 ± 0⋅06	10⋅91 ± 0⋅06	2⋅70 ± 0⋅23	62⋅83 ± 5⋅52	0⋅49 ± 0⋅02
*P*-value*	0⋅553	0⋅135	0⋅063	0⋅035	0⋅363	0⋅265	0⋅355	0⋅844

CI, conicity index; BAI, body adiposity index; AVI, abdominal volume index; BRI, body roundness index; WWI, weight-adjusted-waist index; CMI, cardiometabolic index; LAP, lipid accumulation product; AIP, atherogenic index of plasma.

*P-value < 0⋅05 considered statistically significant (two-tailed).

Testing the correlation between the intake of different types of fat and obesity and coronary indices for the total sample is presented in Table 3. The total fat, SFA, and PUFA intake had a significant moderate correlation with AVI (*r* = 0⋅389, *r* = 0⋅350, and *r* = 0⋅321, respectively) and BRI (*r* = 0⋅351, *r* = 0⋅327, and *r* = 0⋅322; respectively), while had a weak correlation with the rest of the indices (*P* < 0⋅05) except SFA with CMI (*P* = 0⋅074). MUFA intake had a significantly weak correlation with CI, AVI, BRI, WWI, and AIP (*P* < 0⋅05). Chol intake had a significant weak correlation with AVI (*r* = 0⋅156, *P* = 0⋅001), BRI (*r* = 0⋅153, *P* = 0⋅001), and AIP (*r* = 0⋅135, *P* = 0⋅003), whereas it had a significant no correlation with CI (*r* = 0⋅097, *P* = 0⋅031) and WWI (*r* = 0⋅096, *P* = 0⋅034). At the same time, omega-6 fatty acids showed a significantly weak correlation with all the indices (*P* < 0⋅05). However, omega-3 fatty acids showed a significantly weak correlation with all indices except CMI.

**Table 3.** The correlation between the intake of different types of fat and obesity and cardiovascular indices

		CI	BAI	AVI	BRI	WWI	CMI	LAP	AIP
**Total**									
Fat (g)	Pearson *r*	0⋅264**	0⋅193**	0⋅389**	0⋅351**	0⋅231**	0⋅100*	0⋅127**	0⋅172**
	*P*-value	<0⋅001	<0⋅001	<0⋅001	<0⋅001	<0⋅001	0⋅027	0⋅005	<0⋅001
Saturated fatty acids (g)	Pearson *r*	0⋅232**	0⋅221**	0⋅350**	0⋅327**	0⋅210**	0⋅081	0⋅095*	0⋅146**
	*P*-value	<0⋅001	<0⋅001	<0⋅001	<0⋅001	<0⋅001	0⋅074	0⋅036	0⋅001
Monounsaturated fatty acids (g)	Pearson *r*	0⋅163**	0⋅059	0⋅235**	0⋅184**	0⋅118**	0⋅040	0⋅078	0⋅123**
	*P*-value	<0⋅001	0⋅195	<0⋅001	<0⋅001	0⋅009	0⋅377	0⋅084	0⋅006
Polyunsaturated fatty acids (g)	Pearson *r*	0⋅197**	0⋅226**	0⋅321**	0⋅322**	0⋅199**	0⋅105*	0⋅122**	0⋅107*
	*P*-value	<0⋅001	<0⋅001	<0⋅001	<0⋅001	<0⋅001	0⋅021	0⋅007	0⋅018
Cholesterol (mg)	Pearson *r*	0⋅097*	0⋅055	0⋅156**	0⋅153**	0⋅096*	0⋅063	0⋅076	0⋅135**
	*P*-value	0⋅031	0⋅226	0⋅001	0⋅001	0⋅034	0⋅161	0⋅092	0⋅003
Omega-3 fatty acid (g)	Pearson *r*	0⋅182**	0⋅144**	0⋅288**	0⋅262**	0⋅163**	0⋅088	0⋅126**	0⋅115*
	*P*-value	<0⋅001	0⋅001	<0⋅001	<0⋅001	<0⋅001	0⋅051	0⋅005	0⋅011
Omega-6 fatty acid (g)	Pearson *r*	0⋅178**	0⋅215**	0⋅296**	0⋅299**	0⋅181**	0⋅100*	0⋅111*	0⋅099*
	*P*-value	<0⋅001	<0⋅001	<0⋅001	<0⋅001	<0⋅001	0⋅027	0⋅014	0⋅028

CI, conicity index; BAI, body adiposity index; AVI, abdominal volume index; BRI, body roundness index; WWI, weight-adjusted-waist index; CMI, cardiometabolic index; LAP, lipid accumulation product; AIP, atherogenic index of plasma.

** Correlation is significant at the 0⋅01 level (two-tailed).

* Correlation is significant at the 0⋅05 level (two-tailed).

The gender-specific correlation between the intake of different types of fat and obesity and coronary indices is presented in Table 4. For males, the fat intake had a significant moderate correlation with BAI (*r* = 0⋅315, *P* < 0⋅001), AVI (*r* = 0⋅321, *P* < 0⋅001), and BRI (*r* = 0⋅327, *P* < 0⋅001), and a significant weak correlation with CI (*r* = 0⋅162, *P* = 0⋅003) and WWI (*r* = 0⋅173, *P* = 0⋅001). In female participants, the fat intake had a significant moderate association with BAI (*r* = 0⋅385, *P* < 0⋅001), AVI (*r* = 0⋅370, *P* < 0⋅001), and BRI (*r* = 0⋅385, *P* < 0⋅001), and a significant weak association with the rest of the indices (*P* < 0⋅001). For saturated fat, male participants showed a significant moderate correlation with BAI (*r* = 0⋅317, *P <* 0⋅001) and BRI (*r* = 0⋅308, *P* < 0⋅001), and a significant weak correlation with CI, AVI, and WWI (*P* < 0⋅05), while no significant correlation with all of the three studied coronary indices. On the other hand, female participants showed a significant moderate correlation with BAI (*r* = 0⋅413, *P* < 0⋅001), AVI (*r* = 0⋅337, *P* < 0⋅001), and BRI (*r* = 0⋅350, *P* < 0⋅001), and a significant weak correlation with LAP (*r* = 0⋅223, *P* = 0⋅007).

**Table 4.** The correlation between the intake of different types of fat and obesity and coronary indices based on gender (*n* 491)

		CI	BAI	AVI	BRI	WWI	CMI	LAP	AIP
Male (*n* 344)									
Fat (g)	Pearson *r*	0⋅162**	0⋅315**	0⋅321**	0⋅327**	0⋅173**	0⋅011	0⋅044	0⋅062
	*P*-value	0⋅003	<0⋅001	<0⋅001	<0⋅001	0⋅001	0⋅838	0⋅415	0⋅254
Saturated fatty acids (g)	Pearson *r*	0⋅155**	0⋅317**	0⋅289**	0⋅308**	0⋅173**	0⋅009	0⋅025	0⋅076
	*P*-value	0⋅004	<0⋅001	<0⋅001	<0⋅001	0⋅001	0⋅862	0⋅647	0⋅159
Monounsaturated fatty acids (g)	Pearson *r*	0⋅039	0⋅163**	0⋅143**	0⋅146**	0⋅049	−0⋅032	0⋅011	0⋅012
	*P*-value	0⋅476	0⋅002	0⋅008	0⋅007	0⋅368	0⋅554	0⋅846	0⋅831
Polyunsaturated fatty acids (g)	Pearson *r*	0⋅152**	0⋅239**	0⋅294**	0⋅276**	0⋅143**	0⋅025	0⋅057	0⋅029
	*P*-value	0⋅005	<0⋅001	<0⋅001	<0⋅001	0⋅008	0⋅641	0⋅289	0⋅591
Cholesterol (mg)	Pearson *r*	0⋅039	0⋅109*	0⋅113*	0⋅132*	0⋅057	0⋅068	0⋅058	0⋅145**
	*P*-value	0⋅470	0⋅044	0⋅037	0⋅014	0⋅290	0⋅205	0⋅281	0⋅007
Omega-3 fatty acid (g)	Pearson *r*	0⋅106*	0⋅231**	0⋅242**	0⋅223**	0⋅099	0⋅036	0⋅076	0⋅071
	*P*-value	0⋅050	<0⋅001	<0⋅001	<0⋅001	0⋅067	0⋅505	0⋅158	0⋅190
Omega-6 fatty acid (g)	Pearson *r*	0⋅140**	0⋅233**	0⋅282**	0⋅264**	0⋅131*	0⋅019	0⋅048	0⋅021
	*P*-value	0⋅009	<0⋅001	<0⋅001	<0⋅001	0⋅015	0⋅727	0⋅372	0⋅692
Female (*n* 147)									
Fat (g)	Pearson *r*	0⋅199*	0⋅385**	0⋅370**	0⋅385**	0⋅215**	0⋅201*	0⋅284**	0⋅190*
	*P*-value	0⋅016	<0⋅001	<0⋅001	<0⋅001	0⋅009	0⋅015	<0⋅001	0⋅021
Saturated fatty acids (g)	Pearson *r*	0⋅149	0⋅413**	0⋅337**	0⋅350**	0⋅160	0⋅156	0⋅223**	0⋅104
	*P*-value	0⋅071	<0⋅001	<0⋅001	<0⋅001	0⋅053	0⋅059	0⋅007	0⋅211
Monounsaturated fatty acids (g)	Pearson *r*	0⋅095	0⋅269**	0⋅236**	0⋅244**	0⋅106	0⋅100	0⋅169*	0⋅138
	*P*-value	0⋅252	0⋅001	0⋅004	0⋅003	0⋅202	0⋅229	0⋅041	0⋅095
Polyunsaturated fatty acids (g)	Pearson *r*	0⋅279**	0⋅312**	0⋅368**	0⋅390**	0⋅298**	0⋅264**	0⋅338**	0⋅230**
	*P*-value	0⋅001	<0⋅001	<0⋅001	<0⋅001	<0⋅001	0⋅001	<0⋅001	0⋅005
Cholesterol (mg)	Pearson *r*	0⋅129	0⋅098	0⋅185*	0⋅183*	0⋅131	−0⋅006	0⋅082	0⋅019
	*P*-value	0⋅120	0⋅236	0⋅025	0⋅026	0⋅113	0⋅946	0⋅323	0⋅815
Omega-3 fatty acid (g)	Pearson *r*	0⋅232**	0⋅196*	0⋅311**	0⋅318**	0⋅237**	0⋅158	0⋅247**	0⋅106
	*P*-value	0⋅005	0⋅018	<0⋅001	<0⋅001	0⋅004	0⋅056	0⋅003	0⋅203
Omega-6 fatty acid (g)	Pearson *r*	0⋅250**	0⋅285**	0⋅324**	0⋅352**	0⋅274**	0⋅271**	0⋅326**	0⋅235**
	*P*-value	0⋅002	<0⋅001	<0⋅001	<0⋅001	0⋅001	0⋅001	<0⋅001	0⋅004

CI, conicity index; BAI, body adiposity index; AVI, abdominal volume index; BRI, body roundness index; WWI, weight-adjusted-waist index; CMI, cardiometabolic index; LAP, lipid accumulation product; AIP, atherogenic index of plasma.

** Correlation is significant at the 0⋅01 level (two-tailed).

* Correlation is significant at the 0⋅05 level (two-tailed).

For both genders, significant weak correlations were found between Chol consumption and AVI (*r* = 0⋅113, *P =* 0⋅037; *r* = 0⋅185, *P =* 0⋅025; males and females, respectively), and BRI (*r* = 0⋅132, *P =* 0⋅014; *r* = 0⋅183, *P =* 0⋅026; males and females, respectively). Among both male and female participants, MUFA intake had a significant weak correlation with BAI (*r* = 0⋅163, *P =* 0⋅002; *r* = 0⋅269, *P* = 0⋅001, respectively), AVI (*r* = 0⋅143, *P =* 0⋅008; *r* = 0⋅236, *P* = 0⋅004, respectively), and BRI (*r* = 0⋅146, *P =* 0⋅007; *r* = 0⋅244, *P* = 0⋅003, respectively). Female participants additionally showed a weak correlation between MUFA intake and LAP (*r* = 0⋅169, *P =* 0⋅041).

Regarding PUFA and omega-6 fatty acids intake, male participants’ intake of both fats showed a significantly weak correlation with CI, BAI, AVI, BRI, and WWI. In comparison, female participants’ intake from the same fat types showed a significantly weak correlation with CI, WWI, CMI, and AIP and a significantly moderate correlation with AVI, BRI, and LAP. Regarding BAI, there was a significant weak correlation between omega-6 fatty acids intake and a significant moderate correlation with PUFA (*P* < 0⋅05). Omega-3 intake in male participants had a significantly weak correlation with CI, BAI, AVI, and BRI, whereas in females had a significant moderate correlation with AVI and BRI and a significantly weak correlation with CI, BAI, WWI, and LAP (*P* < 0⋅05).

## Discussion

Jordanian population's traditional dishes and the most frequently consumed foods all contain a lot of fat. All meals presented within households usually have a variety of fried foods consumed daily by all family members. This is not the whole picture. Also, there is heavy use for the ‘added spread-fat’, frequently used as a seasoning or flavouring agent, and most Jordanians consume olive oil as one of the main dishes eaten daily with different types of bread for breakfast, dinner, or even as snacks. These eating habits are consistent with the high percentage of consumed fat detected in the current study (39 %). A previous study conducted among Jordanians in 2014 found that the percentage of total fat intake was 26⋅6 %.^(35)^ Our finding is matched with a prospective cohort study that lasted 25 years. Researchers found that total fat intake increased in males and females; they reported an increase in fat intake among females from 22⋅8 % in 1991 to 36⋅3 % in 2015 while in males from 22⋅2 to 35⋅5 %, respectively.^(36)^ As well as, Julibert and others found that all of their participants were less likely to meet the recommendations for fat (mean intake >35 % of total energy intake).^(37)^

In the present findings, the total fat, SFA, and PUFA intake had a significant moderate correlation with AVI and BRI while having a weak correlation with the rest of the indices. However, a large evidence base reported a weak to no correlation between total fat consumption and NCD morbidity and mortality.^(38–40)^ Thus, the present results of excessive total fat intake (39 % *v*. 35 % = AMDR upper limit) are not the only reason for elevated cases of NCDs in Jordan.^(1)^ Fat quality is the key. Thus, focus on individual fat types should be framed as expected when studying dietary fat. This is not new; many agencies and organisations have changed their goals and efforts to highlight the importance of adopting a healthy diet with a limited amount of total fat and a well-known quantity or percent of certain types of fat.^(1)^ Approximately 40 % of the present study population consumes higher than recommended dietary cholesterol (>300 mg/d). This is consistent with the daily *per capita* estimated cholesterol intake among Jordanians, which was 303 mg in 2010,^(35)^ while 204 mg/d in 2006/2007.^(41)^ These findings present a trend of increased cholesterol consumption with advancing time among the Jordanian population and ringing the alarm of dietary change towards an unhealthy/obesogenic diet type. This type of diet is nutrient-deficient and correlated with increased weight, consequently increasing the risk of NCD morbidity and mortality rates.^(2,42)^

Most of the recent literature studied the SFA specifically to identify its effect on health and to pinpoint which fat type is the worst. We found that around half of our study participants (47⋅1 %) consumed more than the recommended SFA (>10 % of total energy intake). González-Becerra and his colleagues found that specific types of SFAs have been associated with the presence or development of obesity, T2DM, proinflammatory profile, atherosclerosis, and insulin resistance.^(43)^

As a result of the belief that total fat plays a significant role in the development of NCDs and their related risk factors, primarily obesity, decades of dietary guiding messages suggest limiting total fat in the diet. A considerable body of evidence combines overweight/obesity development with fat overconsumption.^(17,20,26,44)^ Moreover, many studies reported a significant association between a specific type of fat and obesity.^(19,45,46)^ The correlations between relative proportions of fatty acids and obesity/overweight determined by indices are lacking despite extensive research on the relationships between obesity and total fat and different types of fat. This study evaluated associations between dietary fat types and specific obesity and coronary indices.

Recently, most epidemiological studies worldwide shifted towards using different anthropometric indices to pinpoint the truth about body weight.^(5,6,40,47)^ After the debate about using only BMI to define overweight/obesity.^(2)^ Górnicka and his colleagues reported that the measurement of body fat content should become a generally accepted indicator for effective diagnosis and screening of obesity.^(48)^ The robustness of the studies, in terms of BMI and WC and their correlation with total fat and different fat types, is undoubted. However, in the current study, the total fat intake had a significant moderate correlation with AVI and BRI while having a weak correlation with the rest of the indices. Similarly, Suara and his colleagues reported a positive association between the atherogenicity index and fat percentage from total energy intake.^(46)^

However, the association between obesity and coronary indices with consumption of dietary fat types is unclear, and few studies demonstrate a part of it. Moreover, this was not been studied before among the Jordanian population. Participants who consumed more than the recommended amount of cholesterol among our study population were found to have higher CI, AVI, BRI, and WWI scores than an acceptable amount of consumers, and they also had a lower score of AIP. Furthermore, cholesterol intake had a significantly weak correlation with those indices among the whole study population. In the gender-specific analysis, both genders only had significantly weak correlations with AVI and BRI. At the same time, BAI and AIP were found to be significantly correlated with cholesterol intake among males. However, Kohansal and his colleagues found a significant direct association between plant proteins and BRI and documented that more plant protein consumption was related to higher CI.^(49)^ In contrast, plant proteins are cholesterol-free as the cholesterol is from animal origin. Moreover, vegetarian females showed better body composition and dietary quality than omnivorous females. Body composition was studied by calculating AVI, LAP, and BRI.^(50)^

The AIP is an important predictor of atherosclerosis and CVD risk; it is also superior to the standard atherosclerotic lipid profile.^(15)^ Caminhotto and others documented a CVD risk reduction; i.e. AIP scores decreased from 58⋅3 to 33⋅3 % following the Atkins diet to reveal that this type of diet could significantly reduce the AIP in overweight adult males.^(51)^ In further investigation, Shin and colleagues examined the relationship between AIP and obesity indices and nutrient intake status; they found that as the level of AIP increased, intake of SFA, milk, and dairy products decreased significantly, and the contribution rate of milk and dairy products to fat intake decreased.^(15)^ Interestingly, the Atkins diet, as well as milk and dairy products, all have a considerable amount of cholesterol, so the aforementioned correlations supported our inverse result between AIP scores and cholesterol consumption; it also highlighted the gender-specific association as both studies were conducted among men. Furthermore, a direct relationship between dietary fat quality, increased BMI, and lipid abnormalities with AIP has been approved.^(14)^

Also, we found that mean BRI scores only significantly differ among omega-6 consumption, where acceptable intake consumers had a higher BRI score (1⋅9) than unacceptable intake consumers (1⋅7). While omega-6 fatty acids showed a significantly weak correlation with all the indices among the total analysis of the study population, regarding gender differences, male participants’ intake showed a significantly weak correlation with CI, BAI, AVI, BRI, and WWI. At the same time, female participants’ intake showed a significantly weak correlation with CI, BAI, WWI, CMI, and AIP and a significantly moderate correlation with AVI, BRI, and LAP. Omega-6 fatty acid intake increased, and omega-3 fatty acid intake decreased over the past three decades in Western diets, resulting in a significant increase in the omega-6/omega-3 ratio from 1:1 during evolution to 20:1,^(52)^ or it might be higher today. Consequently, the prevalence of overweight and obesity has significantly increased, paralleled by a shift in the content of fatty acids.

However, omega-3 fatty acids in our analysis showed a significantly weak correlation with all indices except for CMI without a significant difference between recommended intakes’ consumers and non-consumers. Meanwhile, omega-3 intake in male participants had a significantly weak correlation with CI, BAI, AVI, and BRI. In contrast, females had a significantly moderate correlation with AVI and BRI and a significantly weak correlation with CI, BAI, WWI, and LAP. Garaulet and his colleagues reported that central obesity (defined by WC and CI) was positively associated with omega-6 and inversely associated with MUFA and omega-3 in adipose tissue.^(53)^ Although our correlations were positive with the studied indices, either obesity or coronary, we did not check the ratio between omega-6 and omega-3 fatty acids. Weta and his colleagues, in an interventional study, reported that the reduction of body fat could be enhanced by supplementation with a high dose of a low omega-6/omega-3 ratio; they documented a significant reduction of BMI, WC, CI, and LAP within the interventional group.^(54)^

Extensively, a large base of evidence recommends a balanced intake of omega-3 and omega-6 to reduce the risk of obesity and its related NCDs.^(19,52)^ Furthermore, Suara and his colleagues suggested that for obesity prevention, it is essential to increase the intake of omega-3/omega-6 to higher ratios.^(46)^ PUFA intake, which mainly consists of omega-3 and omega-6, had a significant moderate correlation with AVI and BRI, while it had a weak correlation with the rest of the indices. Male participants’ intake significantly weakly correlated with CI, BAI, AVI, BRI, and WWI. In comparison, female participants’ intake showed a significantly weak correlation with CI, WWI, CMI, and AIP and a significantly moderate correlation with BAI, AVI, BRI, and LAP. A prospective cohort study by Beulen and his colleagues found that replacing 5 % energy from SFA with MUFA or PUFA resulted in weight changes of −0⋅38 and −0⋅51 kg, respectively.^(19)^ In comparison, Brayner and others conclude that a dietary pattern with higher SFA and lower PUFA foods was associated with obesity and abdominal obesity.^(45)^ In this study, MUFA intake had a significantly weak correlation with CI, AVI, BRI, WWI, and AIP. In line with these findings, Mohit and colleagues (2022) approved that MUFA levels of more than 12 % of calories in weight loss diets have been significantly associated with a further reduction of adipose tissue; they also found a significant inverse relationship between WC and CI with PUFA/SFA and MUFA/SFA levels, they also revealed that by substituting MUFA and PUFA for SFA, one can minimise the buildup of adipose tissue and preserve more lean body mass.^(55)^ However, the current study revealed that SFA intake had a significant moderate correlation with AVI and BRI while having a weak correlation with the rest of the indices except CMI. Moreover, Brayner and others found that higher SFA foods were associated with obesity and abdominal obesity.^(45)^

We tried in our analysis to differentiate between the two types of indices (obesity and coronary) to identify the best way to explain the whole figure about different fat types, either purely anthropometric or containing some biochemical data. Most fat types did not correlate to coronary indices, except Aortic pulsatility index. Since numerous non-linear relationships exist between dietary intake and anthropometric, coronary, or health outcomes, dietary findings must be corrected for total energy intake.

Curiously, to establish a balanced diet and lifestyle and collectively lower illness risk, simultaneous, effective public health interventions are needed. The worldwide obesity crisis is more than fat quality alone. Contrary to popular belief, the current result revealed that it is not necessary to restrict fat in our diets to prevent chronic diseases. The type of fat is more relevant than the overall amount of fat. Further work is recommended to confirm the role of type of fat in obesity development and, consequently chronic diseases.

The outcomes of this study might be limited by using a cross-sectional design, as the causal relationships between obesity and coronary indices and the dietary intake of types of fat could not be adequately confirmed. In addition, the omega-6/omega-3 ratio was not calculated, there was a lack of information on fat cooking, and the sample size was relatively small. However, to the best of our knowledge, this is the first study that examines the relationship between both obesity and coronary indices and the consumption of dietary fat from different types among Jordanian adults. These findings hope to highlight the relationship between the Jordanian population's dietary habits and the high prevalence of NCD to develop programmes aimed at educating and counselling individuals with risk and also about healthy behaviour modification and changing and replacing recent habits with new healthier ones.

## Conclusion

The total fat intake and different fat types had other correlations with obesity and coronary indices. The total fat, SFA, and PUFA intake significantly correlated with AVI and BRI. MUFA and cholesterol intake had a significantly weak correlation with CI, AVI, BRI, WWI, and AIP. However, omega-3 fatty acids showed a significantly weak correlation with all indices except CMI. All the abovementioned correlations depend on fat-type and gender-specific correlations.
